# A comparison between whole transcript and 3’ RNA sequencing methods using Kapa and Lexogen library preparation methods

**DOI:** 10.1186/s12864-018-5393-3

**Published:** 2019-01-07

**Authors:** Feiyang Ma, Brie K. Fuqua, Yehudit Hasin, Clara Yukhtman, Chris D. Vulpe, Aldons J. Lusis, Matteo Pellegrini

**Affiliations:** 1 Molecular Biology Institute, University of California, Los Angeles, California USA; 2 Department of Medicine, Cardiology, University of California, Los Angeles, California USA; 3 Department of Physiological Sciences, University of Florida, Gainesville, Florida USA; 4 Department of Human Genetics, University of California, Los Angeles, California USA; 5 Department of Microbiology, Immunology and Molecular Genetics, University of California, Los Angeles, California USA; 6 Department of Bioinformatics, University of California, Los Angeles, California USA

**Keywords:** Traditional RNA-Seq, 3’ RNA-Seq, Iron metabolism, Gene expression

## Abstract

**Background:**

3’ RNA sequencing provides an alternative to whole transcript analysis. However, we do not know a priori the relative advantage of each method. Thus, a comprehensive comparison between the whole transcript and the 3′ method is needed to determine their relative merits. To this end, we used two commercially available library preparation kits, the KAPA Stranded mRNA-Seq kit (traditional method) and the Lexogen QuantSeq 3’ mRNA-Seq kit (3′ method), to prepare libraries from mouse liver RNA. We then sequenced and analyzed the libraries to determine the advantages and disadvantages of these two approaches.

**Results:**

We found that the traditional whole transcript method and the 3’ RNA-Seq method had similar levels of reproducibility. As expected, the whole transcript method assigned more reads to longer transcripts, while the 3′ method assigned roughly equal numbers of reads to transcripts regardless of their lengths. We found that the 3’ RNA-Seq method detected more short transcripts than the whole transcript method. With regard to differential expression analysis, we found that the whole transcript method detected more differentially expressed genes, regardless of the level of sequencing depth.

**Conclusions:**

The 3’ RNA-Seq method was better able to detect short transcripts, while the whole transcript RNA-Seq was able to detect more differentially expressed genes. Thus, both approaches have relative advantages and should be selected based on the goals of the experiment.

**Electronic supplementary material:**

The online version of this article (10.1186/s12864-018-5393-3) contains supplementary material, which is available to authorized users.

## Background

High-throughput RNA-sequencing (RNA-Seq) is a powerful tool to characterize and quantify transcriptomes, and is now widely used in biomedical research. RNA-Seq is primarily used to quantify the abundance and relative changes in gene expression across sample groups [1]. It enables a relatively unbiased analysis of the transcriptome, and has single base pair resolution, a wide dynamic range of detection, and low background noise [2]. Moreover, the cost of RNA-Seq is continuously dropping as the cost of sequencing decreases, enabling varied investigations of molecular biology in a more precise and comprehensive manner than is possible with competing technologies [1].

**Fig. 1:**
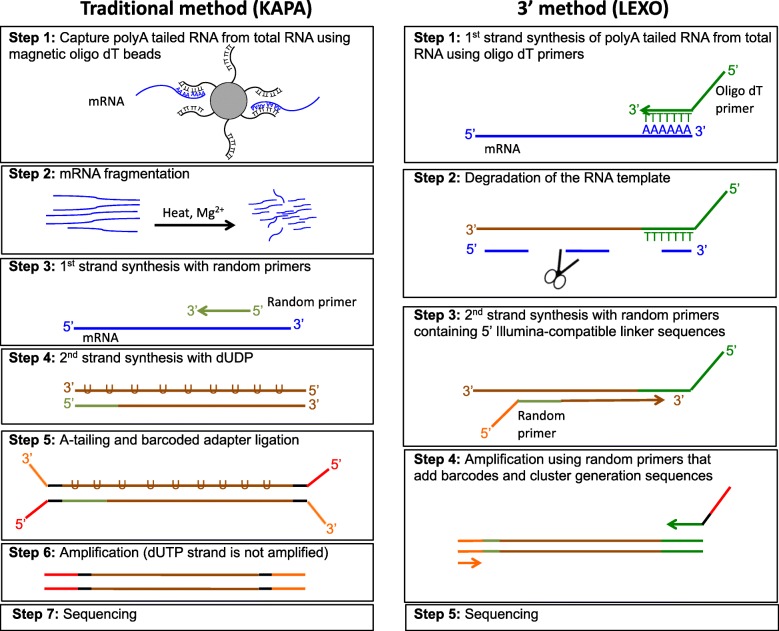
Key library preparation steps for the Trad-KAPA (left) and 3’-LEXO (right) methods

Since the initial application of RNA-Seq, many library preparation methods and sequencing platforms have been established, resulting in a number of choices for users. In the classic whole transcript method, extracted mRNAs are first randomly sheared into fragments, which are then reverse transcribed into cDNAs (Fig. 1). Although RNA-Seq is generally considered unbiased, it is important to note that fragmentation and library construction can introduce some biases into RNA-Seq results [2]. As cDNA fragments are sequenced, the number of reads corresponding to each transcript is proportional to the number of cDNA fragments rather than the number of transcripts. Since longer transcripts are generally sheared into more fragments, more reads will be assigned to them than shorter transcripts. Consequently, when carrying out differential expression analysis, the differentially expressed genes are more likely to be enriched for longer than shorter transcripts, as the statistical power is higher for longer transcripts due to the larger counts [3]. Recently, new 3’ RNA-Seq methods, such as Tag-seq [4] and QuantSeq [5], have been developed to minimize this bias. In the 3’ RNA-Seq method, mRNAs are not fragmented before reverse transcription. Instead, the cDNAs are only reverse transcribed from the 3′ end of the mRNAs, and only one copy of cDNA is generated for each transcript (Fig. 1). Thus, when the cDNAs are sequenced, the number of reads directly reflects the number of transcripts of a certain gene, and the longer and shorter transcripts should have the same coverage of reads.

Since the establishment of 3’ RNA-Seq, it has been used in many studies. For example, Meyer et al. used Tag-Seq to profile gene expression responses of coral larvae [4], Barbash et al. used QuantSeq to quantify gene expression in the human brain [6], and Oberlin et al. used QuantSeq in a genome-wide transcriptome and translatome analysis of Arabidopsis transposons [7]. In all the above-mentioned studies, the genome of the organism that was studied (coral, human and Arabidopsis) was already characterized. However, when little genomic information is available for the species, Tandonnet et al. found that classic RNA-Seq methods worked better than 3’ RNA-Seq methods in quantifying the transcriptome [8].

To determine whether to use the classic whole transcript RNA-Seq method or the 3′ method for a large mouse study where the primary goal is to identify expression quantitative trait loci, we used both methods to prepare RNA-Seq libraries from the livers of mice on two diets, an iron-loaded diet and a control diet. We used the KAPA Stranded mRNA-Seq Kit (Trad-KAPA) to prepare libraries using the whole transcript method, and the Lexogen QuantSeq 3’mRNA-Seq Library Prep Kit-FWD (3’-LEXO) to prepare 3′ libraries. We then sequenced the libraries on the Illumina platform. The sequencing results for the Trad-KAPA and 3’-LEXO libraries were compared to determine their relative advantages and disadvantages. We first mapped the reads to the mouse genome, and confirmed that the Trad-KAPA reads covered the whole transcript, while 3’-LEXO reads only covered the 3′ end. Next, we determined the number of reads assigned to transcripts with different lengths and then used subsampling to determine how sequencing depth affects the read distributions. We also compared the reproducibility of the two methods, and carried out differential expression analysis for both methods.

## Results

### Library preparation and RNA-sequencing

We extracted RNA from the large lobe of the liver from 3 mice on an iron-loaded diet and 3 mice on an iron sufficient control diet and then prepared RNA-Seq libraries using both the Trad-KAPA and 3’-LEXO methods for all six samples. An overview of the key library preparation steps for the two methods are described in Fig. 1. After library preparation, we pooled and sequenced the libraries using single-end sequencing with 50 bp reads on an Illumina HiSeq4000 instrument (Illumina, San Diego, CA). We obtained an average of 22.9 million and 18.4 million reads for Trad-KAPA and 3’-LEXO libraries, respectively. The reads were mapped with STAR 2.5.3a [9] to the mouse genome (mm10 / GRCm38). 80% of the Trad-KAPA reads and 82% of the 3’-LEXO reads were uniquely mapped. As the percentages of mapped reads from the two methods were similar, we randomly sampled 10 million uniquely mapped reads in each sample for further analysis, to make sure that each library had the same sequencing depth.

### 3’-LEXO reads mapped to the 3′ region

**Fig. 2:**
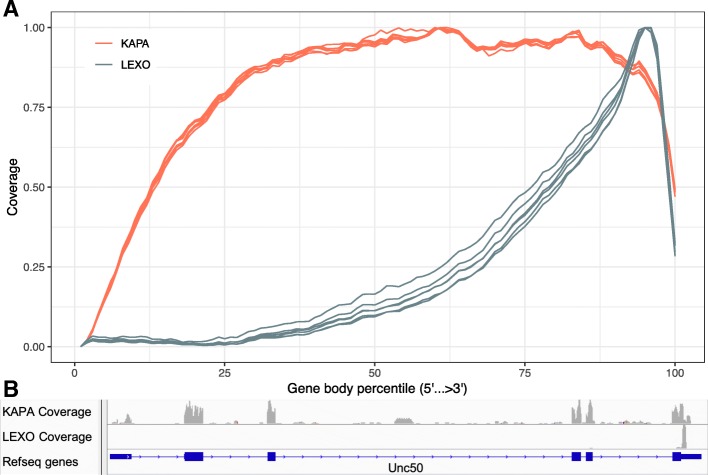
Gene body coverage. **a** Gene body coverage from the Trad-KAPA and 3’-LEXO libraries. **b** Unc50 gene body coverage from the Trad-KAPA and 3’-LEXO libraries

After sequencing and read mapping, we used RSeQC [10] to determine the distribution of the reads along transcripts. As expected, Trad-KAPA reads covered transcripts uniformly, with only a slight decrease in coverage at the 5′ end (Fig. 2a). By contrast, 3’-LEXO reads preferentially mapped to the 3′ end. This suggests that most of the 3’-LEXO reads originated from the 3′ region of the gene. The individual Trad-KAPA libraries (red lines) had very similar transcript coverage profiles, while the individual 3’-LEXO samples (blue lines) exhibited some variation near the middle of the transcript.

We show an example of the coverage differences between Trad-KAPA and 3’-LEXO in Fig. 2b. The mouse Unc50 gene has 6 exons and encodes an inner nuclear membrane RNA binding protein. We used the integrative genomics viewer [11] to visualize Trad-KAPA and 3’-LEXO read coverage. Trad-KAPA reads covered all the exons uniformly, with only a slight decrease in the 5′ exon. There were also some Trad-KAPA reads that mapped to the introns of Unc50, suggesting that some of the introns are not fully spliced. By contrast, most of the 3’-LEXO reads mapped only to the last exon of the gene.

### Trad-KAPA assigned more reads to longer transcripts

**Fig. 3:**
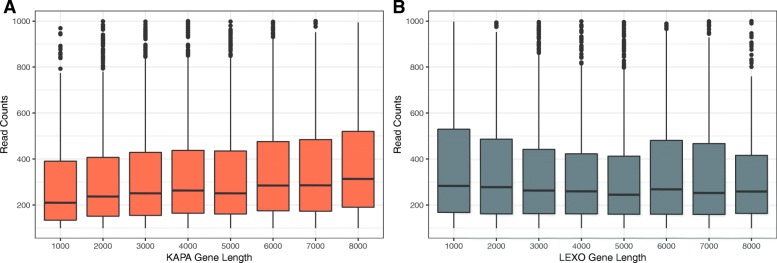
Read counts for transcripts of different length. **a** Trad-KAPA read counts for transcripts with different length. **b** 3’-LEXO read counts for transcripts with different length

Since Trad-KAPA reads originated from the entire transcript while 3’-LEXO reads originated primarily from the 3′ end, we expected that the Trad-KAPA libraries would generate more reads for longer transcripts while the 3’-LEXO libraries would produce equal numbers of reads for transcripts independently of their lengths. To determine whether this is the case, we selected transcripts that have a length range from 500 bp to 8500 bp and have at least 100 read counts, and measured the distribution of coverage levels. For Trad-KAPA libraries, median read counts increased with transcript length (Fig. 3a), indicating that as expected these libraries generate more reads for longer transcripts. By contrast, the median read counts from 3’-LEXO libraries did not change significantly with length (Fig. 3b). This is expected, since the strong 3′ bias found in 3’-LEXO libraries is not significantly affected by transcript length. Thus, for datasets of the same sequencing depth, Trad-KAPA samples contain more reads from longer transcripts, while 3’-LEXO samples appear to be insensitive to transcript length.

### 3’-LEXO recovers more short transcripts as sequencing depth drops

**Fig. 4:**
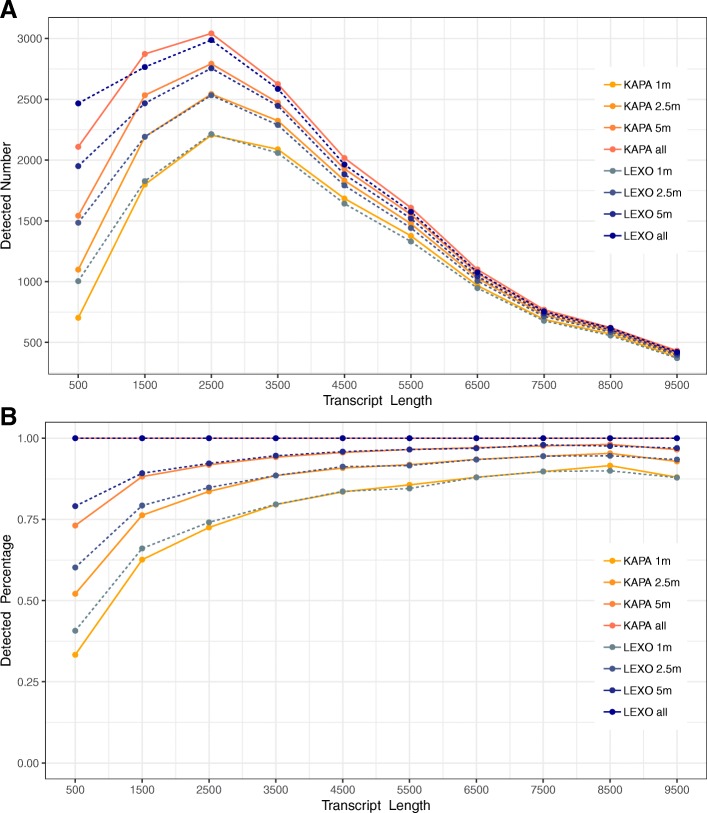
Transcripts of different length detected after subsampling. **a** The number of transcripts of different length detected after subsampling. **b** Percent of transcripts of different length detected after subsampling, compared to sampling at 10 million reads

To determine whether 3’-LEXO detects more short transcripts than Trad-KAPA as sequencing depth drops, we subsampled 1, 2.5 and 5 million uniquely mapped reads for all the samples, and determined how many transcripts with lengths ranging from 0 bp to 10,000 bp were detected (Fig. 4a). As sequencing depth dropped, shorter transcripts were detected less frequently than longer ones in both the Trad-KAPA and 3’-LEXO libraries. When the sequencing depth dropped to 5 million, we found that we detected about 300 more transcripts that are shorter than 1000 bp from the 3’-LEXO libraries than from the Trad-KAPA libraries. With only 2.5 million reads, the difference became even more significant, approaching about 400 transcripts. However, when the sequencing depth dropped to 1 million, the difference became smaller. For transcripts longer than 1000 bp and shorter than 2000 bp, as sequencing depth drops, the detection difference between Trad-KAPA and 3’-LEXO inverted, with 3’-LEXO libraries leading to the detection of slightly more transcripts. For transcripts longer than 2500 bp, while Trad-KAPA always detected slightly more transcripts than 3’-LEXO at all the sequencing depths, the differences were very small.

We also compared the 1, 2.5 and 5 million read depths to 10 million read depth to see how many transcripts were detected by each method as sequencing depth drops. As shown in Fig. 4b, 3’-LEXO detected 10% more transcripts than Trad-KAPA for transcripts shorter than 1000 bp. For transcripts longer than 1000 bp and shorter than 3000 bp, 3’-LEXO only recovered slightly more than Trad-KAPA. For transcripts longer than 3000 bp, the two methods detected about the same percentage of transcripts.

### Trad-KAPA and 3’-LEXO have similar levels of reproducibility

**Fig. 5:**
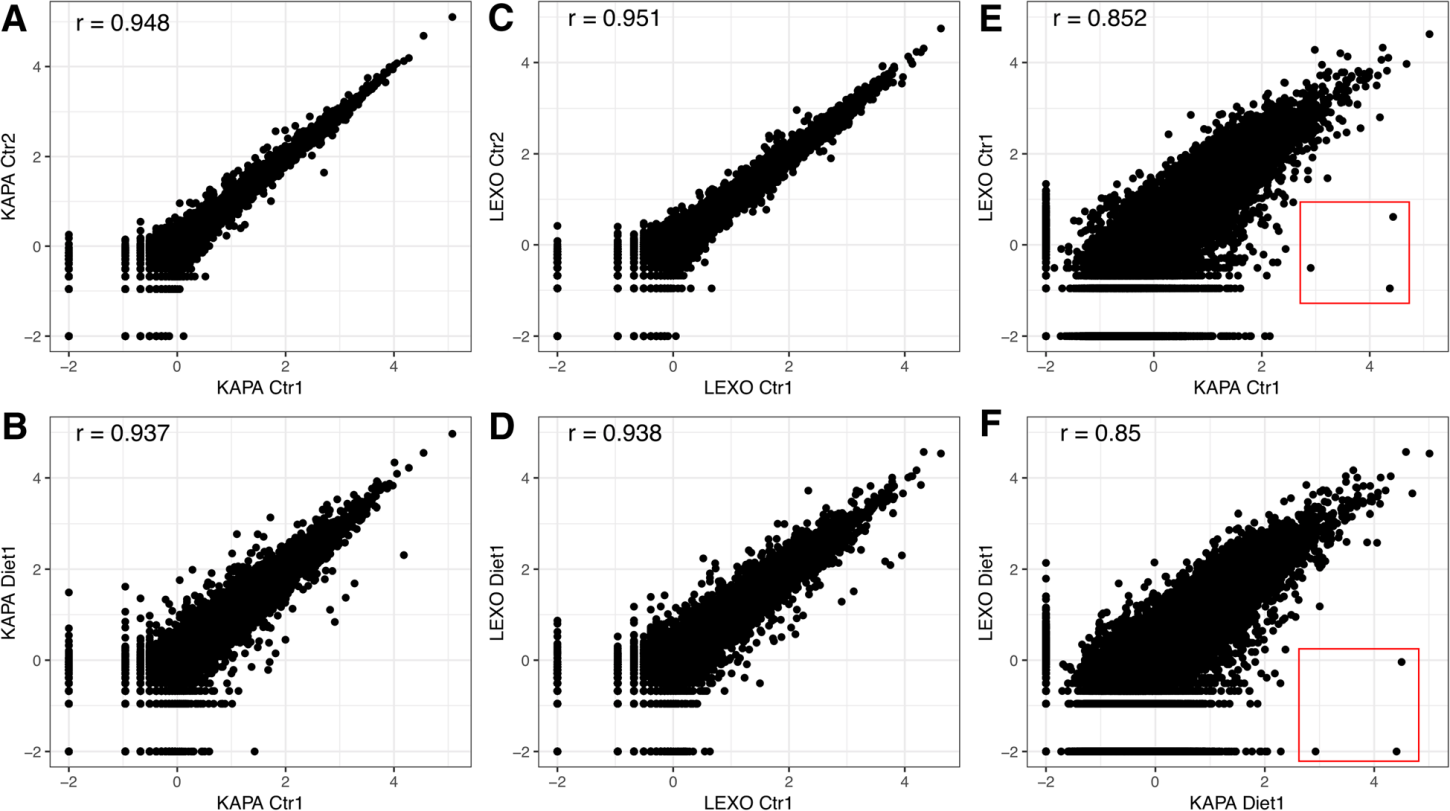
Correlation between Trad-KAPA and 3’-LEXO samples. **a** Correlation between the Trad-KAPA control samples 1 and 2. **b** Correlation between the Trad-KAPA control sample 1 and the iron loaded diet sample 1. **c** Correlation between the 3’-LEXO control samples 1 and 2. **d** Correlation between the 3’-LEXO control sample 1 and the iron loaded diet sample 1. **e** Correlation between the Trad-KAPA and 3’-LEXO control sample 1. **f** Correlation between the Trad-KAPA and 3’-LEXO iron loaded diet sample 1

To compare the reproducibility of the two library preparation methods, we calculated the correlation within and between Trad-KAPA and 3’-LEXO samples. Biological replicates of samples made with each of the two protocols were correlated at comparable levels (Fig. 5a and c), with correlation coefficients around 0.95. The control and diet samples were also highly correlated in both cases (Fig. 5b and d), although slightly lower than that found for the biological replicates. Finally, we also compared libraries generated from the same RNA stock but with the two different library preparation methods (Fig. 5e and f), and found that the correlation coefficient was around 0.85. We found that Trad-KAPA detects some genes that are missed by 3’-LEXO (shown in the red rectangle area in Fig. 5e and f), but generally the agreement between the two libraries was quite high.

### Trad-KAPA detects more differentially expressed genes

One major application of RNA sequencing is the identification of differentially expressed genes (DEGs). We used DESeq2 [12] to carry out differential expression analysis on the control and iron loaded diet samples with subsampling. We adjusted the FDR to 0.05 and detected 1982 and 1157 differentially expressed transcripts for Trad-KAPA and 3’-LEXO, respectively (Table 1). Among those transcripts, 882 were detected by both methods. As sequencing depth drops, the number of differentially expressed transcripts detected by Trad-KAPA and 3’-LEXO decreased, and this trend can also be seen in the MA plots in Additional file 1: Figure S1. However, samples sequenced by Trad-KAPA always resulted in more differentially expressed transcripts when comparing the two libraries at the same sequencing depth. Not surprisingly, more than 95% of the differentially expressed transcripts detected in the subsampled datasets were also detected in the analysis of the initial 10 million read dataset. These results indicate that Trad-KAPA libraries lead to a higher detection of differentially expressed transcripts compared to 3’-LEXO libraries, at all sequencing depths.

**Table 1:** The number of differentially expressed transcripts detected by the Trad-KAPA and 3’-LEXO, before and after subsampling from 10 million reads

Sequencing Depth	Trad-KAPA	Intersection (with 10 m)	3’-LEXO	Intersection (with 10 m)	Intersection (Trad-KAPA and 3’-LEXO)
1 million	343	339 (98.8%)	257	249 (96.9%)	177
2.5 million	758	742 (97.9%)	474	460 (97.0%)	329
5 million	1234	1194 (96.8%)	777	740 (95.2%)	562
10 million	1982	1982	1157	1157	882

The first column denotes the sequencing depth (i.e. the total number of mapped reads from the library examined). The second column denotes the number of differentially expressed transcripts detected by Trad-KAPA. The third column denotes the number of differentially expressed transcripts detected after subsampling that overlap with those from the 10 million sequencing depth. The fourth and fifth columns denote the results for the 3’-LEXO method. The sixth column denotes the number of differentially expressed transcripts detected by both the Trad-KAPA and the 3’-LEXO methods at listed sequencing depth

We also looked at the lengths of the differentially expressed transcripts detected by the two methods. As shown in Additional file 2: Figure S2, some short transcripts were only detected as differentially expressed in 3’-LEXO samples (blue bins). As the transcript length increases, the number of differentially expressed transcripts detected only by 3’-LEXO drops. By contrast, most of the longer transcripts were only detected as differentially expressed by Trad-KAPA. This may be due to the fact that Trad-KAPA assigned more reads to the longer transcripts, which gained enough statistical power to be detected as differentially expressed.

### Validation of the differential expression analysis

To understand why some genes were only detected as significantly differentially expressed in one method, we selected DEGs (1100 from Trad-KAPA and 275 from 3’-LEXO) and compared their expression and log fold changes across both methods (Additional file 3: Figure S3). We found that most genes had higher expression and larger log fold changes in the method that detected them as significantly differentially expressed compared to the other method. However, we also found that the correlation coefficients for the log fold changes and expression levels are 0.87 and 0.83, indicating that the Trad-KAPA and 3’-LEXO methods overall yield consistent results. We compared the expression level of the DEGs detected in only one method to the expression level of the DEGs that were identified in common by both methods and found that these had on average 36% higher expression than the DEGs detected in only one method. Thus, we think the reason for genes being detected as DEGs in only one method was due to lower expression in the other method. This can be explained by the differences that the two methods use in assigning reads to the genes.

We also used RT-qPCR to examine the expression of a subset of the genes that were found to be detected only by either the Trad-KAPA or 3’-LEXO method (mean expression across all six samples [control and iron loaded] > 10 by one method and < 1 by the other). We tested 11 genes that were only detected by the 3’-LEXO method, and 7 genes that were only detected by the Trad-KAPA method (Table 2). Of note, for some of these genes with several reported splice variants, we used multiple primer sets but obtained similar results. For most of these genes, differential expression analysis gave different results for the two RNA-Seq methods. The crossing point-PCR-cycle (Cp) values for 3 of the 3’-LEXO only genes were greater than 30. Of the 8 tested 3’-LEXO only genes that had Cp values less than 30, 5 genes’ RT-qPCR fold change results comparing iron loaded to control diet agreed better with the 3’-LEXO results, 2 agreed better with the Trad-KAPA results, and 1 gave an intermediate result. All of the RT-qPCR results from the 7 tested Trad-KAPA only genes agreed better with the Trad-KAPA results. Thus, as expected, genes that were more highly detected by one method tended to give differential expression results that better agreed with RT-qPCR results.

**Table 2:** RT-qPCR results

Gene name	Primer set used	RT-qPCR fold change	Trad-KAPA fold change	3’-LEXO fold change	RT-qPCR result match which RNA-Seq method	Group
Adnp	mAdnp-ex2–3	0.83	1.15	5.02	Trad-KAPA	Trad-KAPA only
Cd7a	mCd7a-ex3–4	0.69	0.79	5.11	Trad-KAPA	Trad-KAPA only
Fv1	mFv1-F169	0.55	0.54	10.48	Trad-KAPA	Trad-KAPA only
Mid1	mMid1ex4–5	0.77	0.53	5.12	Trad-KAPA	Trad-KAPA only
Mid1	mMid1ex8–9	0.83	0.53	5.12	Trad-KAPA	Trad-KAPA only
Mmp28	mMmp28ex2–3	3.24	4.52	8.55	Trad-KAPA	Trad-KAPA only
Unkl	mUnkl-ex5–6	0.75	1.11	5.12	Trad-KAPA	Trad-KAPA only
Unkl	mUnkl-ex2–3	0.90	1.11	5.12	Trad-KAPA	Trad-KAPA only
Zfp647	mZfp647-204ex4–5	0.55	0.42	8.46	Trad-KAPA	Trad-KAPA only
Zfp647	mZfp647-201ex3–4	0.59	0.42	8.46	Trad-KAPA	Trad-KAPA only
Bcl2a1b	mBcl2a1bEx1–2	2.91	1.44	5.76	In between	3’-LEXO only
Hist4h4	mHist4h4	1.83	0.26	0.27	Neither	3’-LEXO only
Mir5136	mMir5136	1.47	5.07	0.88	3’-LEXO	3’-LEXO only
Mt-Tq	mMt-Tq	1.09	0.95	0.30	Trad-KAPA	3’-LEXO only
Rps27rt	mRps27rt	1.27	0.27	1.40	3’-LEXO	3’-LEXO only
S100a4	mS100a4ex1–2	1.93	1.42	2.31	3’-LEXO	3’-LEXO only
S100a4	mS100a4ex2–3	2.06	1.42	2.31	3’-LEXO	3’-LEXO only
Schip1	mSchip1ex7–8	0.85	0.51	0.90	3’-LEXO	3’-LEXO only
Snord118	mSnord118	0.46	0.26	0.60	3’-LEXO	3’-LEXO only
Snord13	mSnord13	0.92	0.98	0.48	Trad-KAPA	3’-LEXO only
Spink1	mSpink1ex3–4	9.49	2.66	8.28	3’-LEXO	3’-LEXO only
Tceal5	mTceal5ex3–4	5.82	10.48	30.09	Trad-KAPA	3’-LEXO only
Tceal5	mTceal5ex1–2	6.07	10.48	30.09	Trad-KAPA	3’-LEXO only
Atoh8	mAtoh8	3.97	3.10	3.19	Both	Iron metabolism
Bdh2	mBdh2	0.28	0.35	0.39	Both	Iron metabolism
Bmp6	mBmp6	4.83	6.01	6.20	Both	Iron metabolism
Cp	mCp	1.77	1.88	1.94	Both	Iron metabolism
Ftl1	mFtl1	1.75	0.98	3.26	In between	Iron metabolism
Hamp1	mHamp1	5.75	5.19	5.73	Both	Iron metabolism
Hamp2	mHamp2	0.26	0.28	0.33	Both	Iron metabolism
Hfe2	mHfe2	0.61	0.66	0.67	Both	Iron metabolism
Id1	mId1F205&200	4.05	3.43	3.19	Both	Iron metabolism
Lcn2	mLcn2	2.91	2.92	2.25	Both	Iron metabolism
Slc11a2	mSlc11a2	0.66	0.80	0.73	Both	Iron metabolism
Smad7	mSmad7	3.33	3.85	2.91	In between	Iron metabolism
Tfrc	mTfrc	1.16	1.26	1.32	Both	Iron metabolism

Column 3–5 give the log2 fold difference in expression between the iron loaded and control samples by RT-qPCR, Trad-KAPA, and 3’-LEXO. Column 6 indicates if the RT-qPCR results matched better to one RNA-Seq method. Column 7 denotes the group of the genes: detected only in Trad-KAPA (Trad-KAPA only), detected only in 3’-LEXO (3’-LEXO only) or iron metabolism related (Iron metabolism)

### Differential expression in iron metabolism

To validate if the differentially expressed genes detected by each method overlap in terms of biological function, we carried out functional enrichment analyses using the DEGs from both the Trad-KAPA and 3’-LEXO methods using KEGG pathways. We found that the enriched pathways determined from the data from the Trad-KAPA and 3’-LEXO largely overlapped, although there were some pathways specific to each method (Additional file 4: Figure S4A and B). The overlapping pathways were related to amino acid and lipid metabolism. Lipid metabolism in particular has been previously reported to be affected by iron status [13]. We also performed differential expression analysis on previously published microarray data from iron loaded and control C57BL/6 J mice livers [14] and obtained 792 DEGs. We then performed functional enrichment analysis on these DEGs in the same way as for the RNA-Seq results (Additional file 4: Figure S4C). Again, pathways related to amino acid and lipid metabolism were shared between all 3 analyses.

To further determine if the results from both methods were consistent, we examined 13 genes known to be involved in iron metabolism by RT-qPCR, and compared the results with those from both the Trad-KAPA and 3’-LEXO. All 13 genes tested were well represented in both RNA-Seq data sets and had Cp values less than 30 by qPCR. 8 genes were found to have significantly increased expression in the iron loaded livers compared to controls by at least one of the methods (Table 2). Bmp6 and Hamp1 increased 5–6 fold. Atoh8, Smad7, and Id1 increased 3–4 fold. Lcn2 and Cp increased 2–3 fold in all studies. The results for Ftl1 differed between the methods, with Trad-KAPA giving no difference, 3’-LEXO giving a 3 fold increase, and RT-qPCR results about 2 fold increase. The expression of these genes has been reported previously to increase with iron loading [14, 15]. Two tested genes exhibited significantly decreased expression by at least one method. Bdh2 decreased 2–4 fold, and Hamp2 decreased 3–4 fold. The decreased expression of Bdh2 is in agreement with a previous study, but the Hamp2 results (found by all methods) were different than those previously reported for other mouse strains [16]. Finally, 3 genes (Hfe2, Slc11a2, and Tfrc) known to be involved in iron metabolism had little to no difference in expression reported at the mRNA level in the liver with iron loading and also had slight to no differences in expression by the three methods tested here [17, 18]. Thus, the results for both RNA-Seq methods agreed well with both the RT-qPCR results and with previously reported studies.

## Discussion

With the development and advancement of RNA-sequencing technology, many library preparation methods and sequencig platforms have become available. Here, we used a classic whole transcript RNA-Seq method (Trad-KAPA) and a 3’ RNA-Seq method (3’-LEXO) to prepare sequencing libraries from livers of iron-loaded diet and control diet mice, and sequenced the libraries on the Illumina platform. We then compared the sequencing results to determine the advantages and disadvantages of the two approaches.

We identified the gene body coverage of the Trad-KAPA and 3’-LEXO libraries by mapping the reads back to the genome. As expected, Trad-KAPA reads covered transcripts uniformly, with a slight decrease at the 5′ end. One reason for the decrease might be that the secondary structure of the mRNA can cause early termination of reverse transcription [19], making it difficult to reach the cap site (5′ end). It is also possible that many of the transcripts are partially degraded, so that the polyadenylation capture biases the coverage towards the 3′ end. By contrast, 3’-LEXO reads mapped mostly to the 3′ end. 3’-LEXO reads that mapped to the middle of the transcript showed significant coverage variation from library to library. The variation might be caused by the randomness in the reverse transcription start site on the cDNA. In the classic whole transcript method, mRNAs are first sheared into fragments, then the fragments are reverse transcribed to generate cDNAs. Hence, it is expected that the longer a transcript is, the more fragments it should have. The 3’ RNA-Seq method however generates only one read for each transcript, so the number of reads directly reflects the level of gene expression. We counted the reads mapped to transcripts that have lengths ranging from 500 bp to 8500 bp and found that Trad-KAPA libraries had more reads assigned to longer transcripts. By contrast, 3’-LEXO read counts remained uniform as transcript length increased.

As Trad-KAPA assigned more reads to longer transcripts and 3’-LEXO assigned a similar number of reads to transcripts with different lengths, we expected to see fewer short transcripts and more long transcripts detected by Trad-KAPA as sequencing depth drops. For transcripts shorter than 1000 bp, 3’-LEXO detected about 10% more than Trad-KAPA when sequencing depth dropped. However, for transcripts longer than 1000 bp, there was only a small difference between the number detected by Trad-KAPA and 3’-LEXO. Since a 3’ RNA-Seq method only captures reads from the 3′ end of the mRNA, it is difficult for this method to detect differences in isoforms close to the 5′ end of longer genes. In our study, 15% of uniquely mapped Trad-KAPA reads contain splices, while only 6% of uniquely mapped 3’-LEXO reads contain splices. As a result, the 3’ RNA-Seq method is not recommended for novel transcript or splice variant discovery. We also compared Trad-KAPA and 3’-LEXO reproducibility, and found that both methods showed very high reproducibility between biological replicates. When comparing the sequencing results generated with the same mouse using the Trad-KAPA versus 3’-LEXO methods, we found the two methods generally agreed with each other. Although there were a few transcripts detected only by Trad-KAPA, they turned out to be non-coding RNAs.

One major application of RNA-sequencing is to detect differentially expressed transcripts. We subsampled the reads generated by both the methods and carried out differential expression analysis using DESeq2. We found that Trad-KAPA detected more differentially expressed transcripts at all four sequencing depths tested. Interestingly, Xiong et al. [20] also detected more DEGs using the traditional method compared the 3′ method, while Tandonnet et al. [8] detected more DEGs using the 3′ method. We think the differences were caused by removing duplicated reads. Xiong et al. did not remove duplicates in their traditional method but rather used unique molecular identifier to remove the PCR duplicates in their 3′ method. Tandonnet et al. removed all the duplicates in both methods. In our study, we did not remove duplicates, as we believe that instead of PCR over-amplification, the major cause of duplicated reads is very high expression of a small number of genes [21].

Among all the DEGs we found, some of the very short transcripts (shorter than 500 bp) were only detected to be differentially expressed by 3’-LEXO, while many of the long transcripts, especially those longer than 7500 bp, were only detected as differentially expressed by Trad-KAPA. As Trad-KAPA assigns more reads to longer transcripts, the statistical power to detect differences increases. Thus, the probability that those transcripts are detected differentially expressed is higher. It is also clear that as sequencing depth drops, both methods will detect fewer differentially expressed transcripts. Thus, if users want to use RNA-Seq to detect differentially expressed transcripts, Trad-KAPA will likely generate larger lists than 3’-LEXO, biased towards longer transcripts.

## Conclusions

In this paper, we compared two RNA-Seq methods using the classic whole transcript method (Trad-KAPA) and the 3′ method (3’-LEXO). We found that the two methods had similarly high reproducibility between biological replicates. We found that Trad-KAPA assigned more reads to longer transcripts, and thus detected fewer short ones when sequencing depth dropped. However, Trad-KAPA detected more differentially expressed transcripts at all the sequencing depths we tested. With no change of the reproducibility and only slightly better performance in detecting shorter transcripts, but less sensitivity in detecting differentially expressed transcripts, there is no clear advantage to using one method over the other. Thus, we would recommend users select the method based on the goals of their experiments.

## Methods

### Animal husbandry

Eight female SJL/J mice (cat #686, purchased from The Jackson Laboratory, Bar Harbor, ME) housed at 4 mice per cage were placed on an AIN-93G “control” diet containing 50 ppm iron (cat #515005, Dyets, Bethlehem, PA) upon arrival at 3 weeks of age. At 6-weeks of age, one cage of these mice was changed to an AIN-93G “high iron” diet containing 2% carbonyl iron (cat #515007, Dyets). At 11 weeks of age, the mice were fasted starting at 6:30 am, and tissues were collected between 11:30 am and 1 pm. Blood was taken from the retroorbital plexus under isoflurane anesthesia using a heparin-coated capillary tube, and then mice were perfused via the heart with ice-cold phosphate buffered saline to flush remaining blood from the tissues. Tissues were collected and frozen in liquid nitrogen and stored at − 80 °C until analysis.

### Liver RNA purification

Total RNA was extracted from a 20 mg piece of the large lobe of six livers (3 per diet group) using the Qiagen miRNeasy Mini kit (cat# 217004, Qiagen) per the manufacturer’s instructions. In brief, samples were homogenized in QIAzol lysis reagent using a rotor stator homogenizer. Chloroform was added and the extract was vigorously shaken and then centrifuged at 12,000 g to phase separate the organic and aqueous phases. Total RNA was purified from the aqueous phase using the kit spin column. DNA was digested on-column per the manufacturer’s instructions using the RNase-Free DNase Set (cat# 79254, Qiagen). RNA concentration was measured using the Qubit RNA BR Assay (cat# Q10211, Molecular Probes) and RNA integrity was measured with an Agilent 2200 Tapestation instrument using the Agilent RNA ScreenTape and Sample Buffer (cat#5067–5576 and cat#5067–5577, Agilent, Santa Clara, CA). All samples had RINe values greater than 8.

### Library generation

Libraries were prepared from the extracted RNA using two different kits, the QuantSeq 3’mRNA-Seq Library Prep Kit-FWD (cat #15, Lexogen, Vienna, Austria), denoted here as “3’-LEXO”, and the KAPA Stranded mRNA-Seq Kit (cat #KK8421, KAPA Biosystems, Wilmington, MA), denoted here as “Trad-KAPA”, per the manufacturers’ instructions using 1 μg of RNA per library.

For the Trad-KAPA libraries, RNA was heated in a thermocycler for 6 min at 94 °C for the fragmentation step, and KAPA Pure Beads (cat #KK8002, KAPA Biosystems) were used for cDNA capture. For the Trad-KAPA adapter ligation reactions, aliquots of 700 nM stock adapters (prepared from 30 μM original stock, cat #KK8700, KAPA Biosystems) were added to give final adapter concentrations of 50 nM. Ten cycles of library amplification were performed, and the libraries were eluted in 23.5 uL 10 mM Tris-HCl (pH 8). The double stranded DNA concentration was quantified using two methods: the Qubit dsDNA BR Assay Kit (cat #Q32853, Molecular Probes), which gave concentrations ranging from 42.1 to 46.7 ng/ μL, and by the KAPA Library Quantification Kit (cat #KK4824, KAPA Biosystems), which gave values approximately 2.5 higher. The molar concentration of cDNA molecules in the individual Trad-KAPA libraries was calculated from the double stranded DNA concentration (as determined by the KAPA Library Quantification Kit) and the region average size (determined by analyzing each sample on an Agilent 2200 Tapestation instrument using the Agilent D1000 ScreenTape and Sample Buffer (cat#5067–5582 and cat#5067–5583, Agilent, Santa Clara, CA)). Aliquots from each library were diluted to 10 nM cDNA molecules in 10 mM Tris-HCl (pH 8) + 0.01% Tween-20 (cat #P1379-25ML, Sigma, St. Louis, MO), and equal volumes were pooled to make the final pooled library for sequencing.

For the 3’-LEXO libraries, indices from the first two columns of the i7 Index Plate for QuantSeq/SENSE for Illumina adapters 7001–7096 (cat #044, Lexogen) were used, and 11 cycles of library amplification were performed. Libraries were eluted in 22 μL of the kit’s Elution Buffer. The double stranded DNA concentration was quantified using the Qubit dsDNA HS Assay Kit (cat #Q32854, Molecular Probes), and by the KAPA Library Quantification Kit, both which gave similar concentrations for each sample that ranged from 1.7 to 4.3 ng/ μL. The molar concentration of cDNA molecules in the individual 3’-LEXO libraries was calculated from the double stranded DNA concentration and the region average size (determined by analyzing each sample on an Agilent 2200 Tapestation instrument using the Agilent High Sensitivity D1000 ScreenTape and Sample Buffer (cat#5067–5584 and cat#5067–5585, Agilent, Santa Clara, CA)). Aliquots containing an equal number of nmoles of cDNA molecules from each library were pooled to give a pooled library with a concentration of 10 nM cDNA molecules. Per the manufacturer’s advice, the final pool was purified once more (to remove any free primers to prevent index-hopping) by adding 0.9x volumes of PB and proceeding from Step 30 onwards in the QuantSeq User Guide protocol. The library was eluted in 22 μL of the kit’s Elution Buffer.

### Sequencing

The pooled libraries were sequenced in an Illumina HiSeq4000 instrument (Illumina, San Diego, CA).

### Transcript coverage

The reads were mapped with STAR 2.5.3a to the mouse genome (mm10 / GRCm38). After mapping, all 12 BAM files were used as input for RSeQC v2.6.4 to calculate transcript coverage. For visualization of the Unc50 gene coverage, control sample 1 BAM files from Trad-KAPA and 3’-LEXO were visualized in Integrative Genomics Viewer.

### Reads subsampling

We randomly sampled 1, 2.5, 5, and 10 million reads that are uniquely mapped to a gene’s exonic regions from each sample. We considered genes to be detected if they had at least 1 read. The transcript length was calculated by adding the lengths of all the exons from the gene.

### Correlation between Trad-KAPA and 3’-LEXO samples

For comparison between samples sequenced by the same method, raw read counts were modified by the addition of 0.01 before log10 transformation, then Pearson correlation coefficients were calculated between each comparison. For comparisons between Trad-KAPA and 3’-LEXO samples, Trad-KAPA raw read counts were divided by transcript length and multiplied by 1000, then the samples were treated as comparison within one method.

### Differential expression analysis

We used DESeq2 to find differentially expressed transcripts in control diet and iron-loaded diet samples for each sequencing depth. The FDR was adjusted to 0.05, and the other parameters were set to default. The number of overlapping differentially expressed transcripts in Trad-KAPA and 3’-LEXO was calculated. For 1, 2.5 and 5 million reads, the overlap between differentially expressed transcripts in subsampled pools and the initial 10 million read sample was computed. The log fold changes from DESeq2 were used to calculate the correlations between the two methods.

### Real-time quantitative PCR

All primers are listed in Table 2. cDNA for real-time quantitative polymerase chain reaction (RT-qPCR) reactions was prepared with High Capacity cDNA Reverse Transcription Kit (cat# 4368814, Life Technologies) using the same liver RNA stock used for the Trad-KAPA and 3’-LEXO library synthesis. KAPA SYBR FAST qPCR reaction mix (cat# KK4611, Roche) was added with primers and run in triplicate on a LightCycler 480 Instrument (Roche). PCR products gave a strong single peak by melt curve analysis. For each mouse and transcript, housekeeping-normalized expression values were calculated as 2^-(Cp GOI – Cp housekeeper)^, where GOI is the gene of interest and Cp is the cycle number where fluorescence reached a set threshold. Three housekeeping genes (TBP, Beta-actin, and HPRT) were selected to control for variation in cDNA amounts. Students’ t-test was performed for each gene and housekeeper to compare expression levels between the three control and three iron loaded mice, and the average t-test *p*-value across all three housekeepers was calculated. For each gene, housekeeper, and animal, housekeeping-normalized expression values for each gene were then normalized to the average level in animals on the control diet by dividing each housekeeping-normalized expression value by the average control group housekeeping-normalized expression value. These fold change values versus control were then averaged for all three housekeepers used, to give a final average fold change value versus control for each gene.

## Additional files

Additional file 1: **Figure S1.** MA plots showing the differentially expressed transcripts detected by Trad-KAPA and 3’-LEXO with subsampling. (DOCX 1076 kb)

Additional file 2: **Figure S2.** The number of differentially expressed transcripts, grouped by transcript length, detected only by Trad-KAPA (red), only by 3’-LEXO (blue) and by both methods (purple). (DOCX 61 kb)

Additional file 3: **Figure S3.** Comparing DEGs detected in only one method. Genes here are DEGs detected in only KAPA (red) or in only LEXO (blue), log2 fold changes (A) and log2 mean expression (B) are compared between the two methods. (DOCX 2385 kb)

Additional file 4: **Figure S4.** KEGG Pathways enriched by Trad-KAPA (A), 3’-LEXO (B) and Microarray (C) DEGs. (DOCX 123 kb)

## Abbreviations

3’-LEXO: Lexogen QuantSeq 3’ mRNA-Seq
cDNA: Complementary
DEGs: Differentially expressed genes
DNA: Deoxyribonucleic acid
FDR: False discovery rate
mRNA: Messenger RNA
RNA: Ribonucleic acid
RNA-Seq: RNA-sequencing
RSeQC: Quality control of RNA-seq experiments
Trad-KAPA: KAPA Stranded mRNA-Seq
