# Supplementary material for: A comparison between whole transcript and 3’ RNA sequencing methods using Kapa and Lexogen library preparation methods
Source: BMC Genomics. 2019 Jan 7;20:9. doi: 10.1186/s12864-018-5393-3 (PMC6323698; doi:10.1186/s12864-018-5393-3)
Supplement: Supplementary file 1 — Figure S1. MA plots showing the differentially expressed transcripts detected by Trad-KAPA and 3’-LEXO with subsampling. (DOCX 1076 kb) [file 12864_2018_5393_MOESM1_ESM.docx]

**Additional file 1**

**Figure S1** MA plots showing the differentially expressed transcripts detected by Trad-KAPA and 3’-LEXO with subsampling.
