# Supplementary material for: A comparison between whole transcript and 3’ RNA sequencing methods using Kapa and Lexogen library preparation methods
Source: BMC Genomics. 2019 Jan 7;20:9. doi: 10.1186/s12864-018-5393-3 (PMC6323698; doi:10.1186/s12864-018-5393-3)
Supplement: Supplementary file 2 — Figure S2. The number of differentially expressed transcripts, grouped by transcript length, detected only by Trad-KAPA (red), only by 3’-LEXO (blue) and by both methods (purple). (DOCX 61 kb) [file 12864_2018_5393_MOESM2_ESM.docx]

**Additional file 2**

**Figure S2** The number of differentially expressed transcripts, grouped by transcript length, detected only by Trad-KAPA (red), only by 3’-LEXO (blue) and by both methods (purple).
