# Supplementary material for: A comparison between whole transcript and 3’ RNA sequencing methods using Kapa and Lexogen library preparation methods
Source: BMC Genomics. 2019 Jan 7;20:9. doi: 10.1186/s12864-018-5393-3 (PMC6323698; doi:10.1186/s12864-018-5393-3)
Supplement: Supplementary file 3 — Figure S3. Comparing DEGs detected in only one method. Genes here are DEGs detected in only KAPA (red) or in only LEXO (blue), log2 fold changes (A) and log2 mean expression (B) are compared between the two methods. (DOCX 2385 kb) [file 12864_2018_5393_MOESM3_ESM.docx]

**Additional file 3**

**Figure S3** Comparing DEGs detected in only one method. Genes here are DEGs detected in only KAPA (red) or in only LEXO (blue), log2 fold changes (A) and log2 mean expression (B) are compared between the two methods.
