# Supplementary material for: A comparison between whole transcript and 3’ RNA sequencing methods using Kapa and Lexogen library preparation methods
Source: BMC Genomics. 2019 Jan 7;20:9. doi: 10.1186/s12864-018-5393-3 (PMC6323698; doi:10.1186/s12864-018-5393-3)
Supplement: Supplementary file 4 — Figure S4. KEGG Pathways enriched by Trad-KAPA (A), 3’-LEXO (B) and Microarray (C) DEGs. (DOCX 123 kb) [file 12864_2018_5393_MOESM4_ESM.docx]

**Additional file 4**

**Figure S4** KEGG Pathways enriched by Trad-KAPA (A), 3’-LEXO (B) and Microarray (C) DEGs.
